# Barriers and Facilitators for Screening Older Adults on Fall Risk in a Hospital Setting: Perspectives from Patients and Healthcare Professionals

**DOI:** 10.3390/ijerph17051461

**Published:** 2020-02-25

**Authors:** Lotte M. Barmentloo, Manon L. Dontje, Moniek Y. Koopman, Branko F. Olij, Christian Oudshoorn, Johan P. Mackenbach, Suzanne Polinder, Vicki Erasmus

**Affiliations:** 1 Department of Public Health, University Medical Center Rotterdam, Erasmus MC, 3000 CA Rotterdam, The Netherlands; 2 Department of Internal Medicine, University Medical Center Rotterdam, Erasmus MC, 3000 CA Rotterdam, The Netherlands

**Keywords:** fall prevention, screening, implementation, older adults, healthcare professionals, barriers, facilitators

## Abstract

We aimed to gain insight into the barriers and facilitators to fall risk screening of older adults visiting the hospital as experienced by patients and healthcare professionals, and to examine the differences between chronic- and acute-care patients. We invited patients (≥70 years) attending the nephrology and emergency department to participate in the screening. Patients and their healthcare professionals were asked to complete a self-administered questionnaire based on the “Barriers and Facilitators Assessment Instrument”. Differences in barriers and facilitators between acute- and chronic-care patients were examined with chi-square tests. A total of 216 patients were screened, and 103 completed the questionnaire. They considered many factors as facilitators, and none as barriers. Acute-care patients were more positive than chronic-care patients about healthcare worker characteristics, such as knowledge and skills. After screening, patients were more open to receiving advice regarding fall prevention. The 36 healthcare professionals considered program characteristics to be facilitators and mainly factors regarding healthcare worker characteristics as barriers to implementation. For patients, the outpatient setting seemed to be a good place to be screened for fall risk. Healthcare professionals also suggested that program characteristics could enhance implementation. However, healthcare professionals’ mindsets and the changing of routines are barriers that have to be addressed first.

## 1. Introduction

Worldwide, falls and fall-related injuries in older adults are a major public health problem [1]. They can cause a decline in the physical and social functioning of older adults [2] and have a huge economic burden on society [3]. Of all older adults aged 65 years and older, one third experience a fall each year [4,5,6]. According to the European Public Health Association [5], at least 3.8 million older people visit the emergency department (ED) due to a fall-related injury each year. Of those, 1.4 million need further treatment and are admitted to the hospital. In the Netherlands, among adults 80 years and older, deaths due to a fall have increased significantly in recent years. Between 2000 and 2016, crude mortality rate increased from 78.1 (95%CI, 70.4–85.9) to 334.0 (95%CI, 320.9–347.1) per 100,000 older adults [7]. Based on the aging population, the number of falls, fall-related visits to the ED, and fatal falls are expected to increase in the next few decades [5,8]. The risk of falling increases with age, as does the risk of a more severe injury as a result of the fall. The most common fall-related injuries are bone fractures (54%), of which most are hip fractures (17%), followed by superficial injuries (12%) and mild brain injuries (11%) [9].

To decrease the incidence of falls and related injuries, fall prevention interventions that reduce fall risks are paramount. Many studies indicate that identifying people at risk of falling by screening and multifactorial fall-prevention interventions are (cost-)effective methods in the prevention of falls [10,11,12,13]. However, the fact that an intervention has proven to be effective in a study setting does not always ensure its successful implementation in practice [14]. In fact, the rate at which prevention programs are implemented into practice is low [15]. Previous studies have shown that healthcare professionals could play an important role in a successful implementation process [16,17]. Firstly, healthcare professionals could be involved in identifying older adults with a high risk of falling. This is important because people are often not aware of their own fall risk, even when it is high [18,19]. Secondly, they could provide and discuss fall-prevention advice and refer patients to fall-prevention programs.

When implementing fall risk screening and prevention interventions, healthcare professionals might encounter several barriers that could inhibit successful implementation. Most studies on these barriers have been performed within primary care settings, in which providing and discussing advice and referring to fall-prevention programs play important roles. The barriers that are mentioned most often in these settings are a lack of knowledge about existing programs and about other healthcare professionals that could be involved, attributing little importance to fall prevention, and a lack of motivation [20,21]. Little is known about the barriers and facilitators experienced by healthcare professionals when implementing fall-prevention interventions in an in-hospital setting; especially fall-prevention activities targeting outpatients. One study examined barriers to implementing practical guidelines for preventing falls as experienced by nurses in different departments within a hospital. This study found that a lack of knowledge and motivation, the availability of supportive staff, the access to facilities, the health status of the patients, and staff education were the main barriers to implementing fall-prevention guidelines [22].

For the successful implementation of a fall risk screening program in a clinical setting it is necessary to gain knowledge of the relevant barriers and facilitators not only of healthcare providers, but also of patients. Most studies focusing on implementing fall-prevention exercise interventions report barriers such as denial, underestimation of risk, time commitment, the dislike of group programs, fear of falling, and no exercise history [16,23,24]. However, in the context of fall risk screening within the hospital, most of these barriers are not relevant, and it is unknown which barriers patients experience in this setting. Moreover, there could be differences in perceived barriers and facilitators in older adults who are receiving chronic care and older adults receiving acute care. Older adults with one or more chronic diseases have a significantly higher risk of falling in comparison with older adults without chronic diseases [25], which could potentially influence their ideas about participating in a fall risk screening and prevention program.

The barriers and facilitators mentioned above occur mainly within fall-prevention programs in community settings. Knowledge of barriers and facilitators in an outpatient setting, where potential patients with an elevated fall risk are present, is lacking. Furthermore, since the focus of this screening was on implementation of the screening program within a hospital setting in general, this study focused on a chronically ill population instead of a population instead of a population in which a high risk is more clear and already expected, such as orthopedic patients. Therefore the aims of this study were (1) to gain insight into the facilitators and barriers to a fall risk screening program for older adults in a clinical setting as perceived by patients (age ≥ 70 years) and healthcare providers, and (2) to examine the differences in the barriers and facilitators between chronic- and acute-care patients.

## 2. Materials and Methods

### 2.1. Study Design and Population

**Figure 1:**
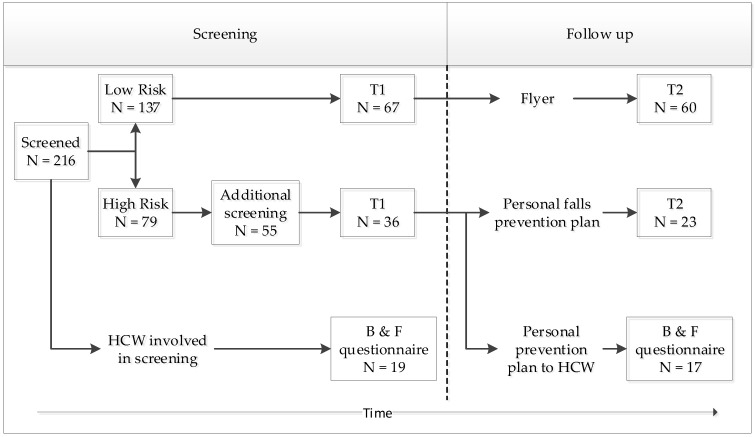
Flowchart of fall risk screening and follow-up. Abbreviations: HCW = healthcare worker; T1 = first questionnaire for patients, which included sociodemographic characteristics and barriers and facilitators; T2 = second questionnaire for patients to assess perceptions about falls and fall prevention; B & F = barriers and facilitators questionnaire for healthcare workers.

This cross-sectional study was part of an implementation study of a fall risk screening program among older adult patients at the Erasmus MC, University Medical Center Rotterdam in the Netherlands. Patients aged 70 years and older who visited the Emergency Department (acute-care patients) or Nephrology outpatient clinic (chronic-care patients) between 1 December 2016 and 31 March 2017 were invited to participate in the fall risk screening program. The study started with a fall risk screening among outpatients. Patients with a low risk of falling received a flyer with their risk status and the advice to visit their general practitioner (GP) when they had any doubts about their fall risk in the future. For patients with an elevated fall risk, an additional comprehensive fall analysis was performed and these patients received a personal fall-prevention plan, which was also sent to their GP (in the case of acute-care patients) or to their nephrologist (in the case of chronic-care patients). The physicians were advised to discuss the results of the screening with their patients and to refer patients to a geriatrician for more comprehensive screening when they deemed this necessary. Patients were asked to complete a questionnaire including questions on sociodemographic characteristics and perceived barriers and facilitators of the screening program within the hospital setting (T1). After three months, patients received another questionnaire regarding their perceptions about falls and fall prevention (T2). Barriers and facilitators of the screening program as perceived by the healthcare professionals at the two departments, as well as the GPs of the patients, were also assessed by questionnaire (see Figure 1). The study protocol was approved by the ethics committee of Erasmus MC (number 2016-666), and informed consent was obtained from all participants.

### 2.2. Screening Program

The fall risk screening program consisted of two steps. The first step was a fall risk test to identify people with an elevated fall risk, which was administered by a nurse of the relevant department. The fall risk test was developed by VeiligheidNL [26] and is primarily used to assess fall risk in community-dwelling older adults (≥65). This simple instrument was developed based on an earlier validated screening tool by Peeters et al. [27], the “Fall Decision Tree” (in Dutch: “de valbeslisboom”). It was developed based on two factors, namely fall history and balance or mobility problems, and is able to identify people with an elevated risk of falling [28,29]. The instrument consists of three questions: (1) “Did you fall during the past twelve months?”, (2) “Do you experience problems with movement and balance?”, and (3) “Are you afraid of falling?” When patients answered “yes” to the first question, or to two out of three questions, patients were considered to have an elevated fall risk. For patients identified as having an elevated fall risk, the fall risk screening program involved a second step. A member of the research team or a trained research nurse contacted these patients by telephone to administer a comprehensive fall risk analysis [26]. This additional and more in-depth analysis included questions about different fall risk domains such as mobility and movement and dizziness, which could give an indication of the fall risk factors for each participant. Based on these factors, a personal prevention plan was set up by the research team and was sent to the patient and to the patient’s GP. Dependent on the risk factors, the prevention plan consisted of exercise programs that were suggested and tips to help with dizziness, painful joints, memory and concentration, vision, and home adjustments. Although the fall risk test has not been officially validated yet, it is the recommended tool for fall risk screening in older adults [28]. An independent committee reviewed the tool and judged the fall risk test to be well substantiated. Therefore, the fall risk test was included in a national database for effective interventions and tools, the “Centre of Healthy Living” (in Dutch: “Loket Gezond Leven”) of the National Institute for Public Health and the Environment (in Dutch: “Rijksinstituut voor Volksgezondheid en Mileu”).

### 2.3. Patient Characteristics

The following sociodemographic characteristics were collected: gender, age, ethnicity, education level, family status (living alone or together with partner and/or children), housing situation (independent, independent with care, or care institution), housing type (ground floor, house or flat with stairs, house or flat with elevator), and chronic conditions (0 or ≥1). Patients were considered Dutch natives when they themselves and both parents were born in the Netherlands. Education level was categorized as low (less than primary school, primary school, and more than primary school but without another diploma), middle (i.e., technical school, vocational education, general secondary/pre-university education), or high (i.e., college/university). For chronic conditions, patients could answer whether they had chronic conditions other than the one for which they visited the hospital. The question consisted of a list of eight chronic conditions (and an option for another condition that was not part of the list).

### 2.4. Barriers and Facilitators

Barriers and facilitators were assessed among patients as well as healthcare providers. The self-administered questionnaires were based on the “Barriers and Facilitators Assessment Instrument” developed by Peters et al. [30]. All statements, for both patients and healthcare professionals, could be divided into the following four domains: (1) fall-prevention program characteristics, (2) healthcare provider characteristics, (3) patient characteristics, and (4) context characteristics. For healthcare professionals, there was an extra domain assessing attitudes. These statements were self-designed based on constructs of the Theory of Planned Behavior [31].

All patients involved in the screening (acute and chronic care) received a baseline questionnaire. For patients with a low fall risk, this questionnaire was assessed directly after case finding, and for patients with a high risk, after the comprehensive analysis. The questionnaire consisted of 17 statements used to assess barriers and facilitators to the fall risk screening program. After three months, patients received a second questionnaire that consisted of nine statements about falls, fall prevention, and fall risk screening to assess perceptions about fall prevention (see Appendix A).

All healthcare professionals that were involved in the fall risk screening and follow-up advice (within the hospital and GPs) received a questionnaire that consisted of 34 statements. The questionnaire for the healthcare professionals that performed the fall risk screening focused on barriers and facilitators for the screening itself; they received the questionnaire one month after all patients were screened. The questionnaire for general practitioners and medical specialists involved in the follow-up focused on the barriers and facilitators of implementing the advice as set up by the research team; they received the questionnaire one month after receiving the prevention advice.

The statements could be answered with a five point Likert scale, with answer categories ranging from 1 (totally disagree) to 5 (totally agree). Using the method described by Peters et al. [30], factors were considered barriers if half or more of the respondents (totally) disagreed with the positive statements, or if half or more of the respondents (totally) agreed with negative statements. Factors were considered facilitators if half or more of the respondents (totally) agreed with the positive statements, or if half or more of the respondents (totally) disagreed with negative statements. In addition, negative statements were recoded and a summary index for each dimension was calculated by dividing the sum score of statements in each dimension by the number of statements. The summary index ranged from 1 (very possibly a barrier) to 5 (very possibly a facilitator).

### 2.5. Data Analysis

For baseline characteristics, dichotomous data were expressed as number and percentages and continuous variables as mean and standard deviation or median and interquartile range (IQR). The differences in baseline characteristics between acute-care and chronic-care patients were tested with chi-square tests for dichotomous data and the Mann–Whitney U test for continuous data. Frequencies of (totally) disagree and (totally) agree were calculated for patients’ and healthcare professionals’ statements, and were expressed as percentages. Chi-square tests were used to test the differences in statements between patients receiving chronic care and patients receiving acute care, and between high- and low-risk patients. In addition, for every dimension, a mean summary index was calculated separately for patients and healthcare professionals. Mann–Whitney U tests were used to compare domains between acute- and chronic-care patients, and between in-hospital healthcare professionals and GPs. In addition, the domains were compared between patients and healthcare professionals. Data were analyzed using SPSS Statistical data software (IBM) version 25 (IBM Corp. Released 2017. IBM SPSS Statistics for Windows, Version 25.0 Armonk. NY: IBM Corp.).

## 3. Results

### 3.1. Sociodemographic Characteristics

In total, 216 patients were screened, of which 116 (53.7%) were chronic-care patients (outpatient clinic) and 100 (46.3%) were acute-care patients (ED). Of those 216, 79 (36.6%) had a high risk of falling. Seventy-seven patients (35.8%) had fallen once or more and 34 (15.8%) had fallen multiple times in the last 12 months. Problems with mobility and balance were experienced by 112 patients (51.9%) and 58 (27.1%) were afraid of falling. The only difference between the departments (chronic vs. acute) was seen in fall history; acute-care patients had fallen more often during the last twelve months (42.2% vs. 28.3%, *p* = 0.033).

Of all patients screened for fall risk, 103 (47.7%) patients completed the barriers and facilitators questionnaire. Of these, 74 were chronic-care patients (response rate: 74%) and 29 were acute-care patients (response rate: 25%). The majority of the respondents was male (73.3%) and the median (IQR) age was 74 (71–77) years. Most patients were born in the Netherlands (89.7%) and lived together with a partner or children without medical help (86.6%). Thirty-five percent of the respondents had a high fall risk. No statistically significant differences between acute-care patients and chronic-care patients were found. More detailed demographic characteristics of these patients are presented in Table 1.

### 3.2. Barriers and Facilitators of Patients

The majority of the patients (both in chronic care and acute care) considered the following factors to be positive factors for implementation of the fall risk screening program: attractiveness of the program, financial status, ethnicity, and time investments. More precisely, the majority of the patients had no negative expectations of fall risk screening (attractiveness) (59.4%), expected to benefit from the screening (attractiveness) (59.8%), were not afraid the screening would cost them money (financial status) (58.0%), had enough time to be screened (time investment) (75.2%), and thought the program was concordant with their culture and/or values (ethnicity) (76.0%). Patients receiving acute care reported more positive factors than patients receiving chronic care. They also considered the skills and knowledge of healthcare workers, the specific healthcare workers involved (specificity/flexibility) the place of screening (facilities), the ideas people in their surroundings had about participating in fall risk screening (group norms), and the need to be screened for fall risk (motivation to change) as positive factors. No statistical differences between departments were seen in percentages that (fully) agreed or (fully) disagreed with statements. The patients did not indicate barriers to the fall-prevention screening program. More information about the statements can be found in Table 2.

Three months after screening for fall risk at the departments, most patients (75.9%) indicated that they were more open to receiving advice on preventing falls. Most patients also indicated that they had a more positive attitude towards fall risk screening and fall prevention (69.9%), and most patients were more inclined to take action to prevent falling (63.9%). No differences were seen between patients who received acute or chronic care. Patients who were at high risk for falls and thus received a personal fall-prevention plan more often reported that they had more knowledge to support prevention of a fall (73.9% vs. 51.7%, *p*-value: 0.044)

### 3.3. Barriers and Facilitators of Healthcare Professionals

Of the healthcare professionals that performed the screening, nineteen completed the questionnaire, including fourteen (73.7%) emergency department professionals (managers, nurses, students, and administrative staff) and 5 (26.3%) healthcare professionals of the nephrology department (healthcare assistants). The facilitators mentioned most frequently were the appropriateness (73.7%), the importance (63.2%), and the attractiveness (63.2%) of the program. However, besides facilitators, healthcare professionals also reported barriers. For healthcare professionals who performed the screening, the most frequently mentioned barriers were patients’ cooperation in applying the program (motivation to change) (84.2%), resistance to working according to protocols (attitude, role perception) (78.9%), and reading and remembering the program (practice involvement) (78.9%). Of the 34 statements, healthcare professionals who performed the screening considered four factors to be facilitators and twelve to be barriers.

Of the healthcare professionals who were involved in follow-up consultation and advice (GPs and medical specialists), seventeen completed the questionnaire. The most mentioned facilitators were the fact that it is not difficult to give preventive care if there are not enough supportive staff (supportive staff) (76.5%), and that is not difficult to give preventive care when not being involved in the setup (setup involvement) (64.7%). Healthcare professionals who were involved in the follow-up reported barriers only within the healthcare worker domain. The barriers mentioned were changing routines in working style (lifestyle, working) (76.5%), resistance to working according to protocols (attitude, role perception) (52.9%), and reading and remembering the program (practice involvement) (52.9%). Of the total of 34 statements, healthcare professionals who were involved in the follow-up considered six factors to be facilitators and three to be barriers. More information about the statements made by healthcare professionals can be found in Table 3.

### 3.4. Similarities and Differences in Domains between Patients and Healthcare Professionals

**Figure 2:**
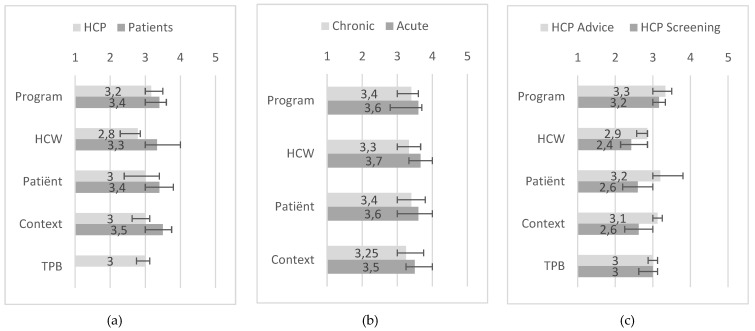
Median and IQR of domains within patients and health care professionals: (**a**) Comparison of healthcare professionals and patients; (**b**) Comparison of chronic and acute care patients; (**c**) Comparison of healthcare professionals that were involved in the advice and that performed the screening. HCP=healthcare professional, GP=general practitioner, HCW=healthcare worker characteristic, TPB=Theory of planned behaviour characteristic.

All statements were summarized into the following domains: program characteristics, healthcare worker characteristics, patient characteristics, and context characteristics. For healthcare professionals, an extra domain regarding statements derived from the Theory of Planned Behavior was added. Patients scored the context characteristics as most probably being a facilitator (median: 3.5, IQR: 3–3.75), while healthcare professionals scored the program characteristics as most probably being a facilitator (median: 3.17, IQR: 3–3.5). Healthcare professionals and patients considered the healthcare worker characteristics to most probably be a barrier. However, this was scored more highly as a barrier by healthcare professionals (median: 2.79, IQR: 2.29–2.86) than by patients (median: 3.33, IQR: 3–4) (*p*-value: <0.001). Other differences between patients and healthcare professionals were seen in context (*p*-value: <0.001) and patient characteristics (*p*-value: <0.001). These domains were scored more highly as possible facilitators for implementing fall risk screening among patients than among healthcare professionals (Figure 2a). Within patients, acute-care patients reported higher scores in the healthcare worker domain than chronic-care patients (*p*-value: 0.016) (Figure 2b). For healthcare professionals, in the domains healthcare worker characteristics (*p*-value: 0.009), patient characteristics, (*p*-value: 0.001), and context characteristics (*p*-value: >0.001), higher scores were reported by healthcare professionals who were involved in the follow-up than by the healthcare professionals who performed the screening (Figure 2c).

## 4. Discussion

The results of this study showed that most patients see no barriers for fall risk screening within an outpatient setting. Overall, they perceived multiple facilitators for implementing fall risk screening, and no barriers. Most patients perceived the screening to be concordant with their ethnic values and as a small time investment. Older adults presenting at the ED identified more facilitators compared to patients with a chronic health problem. Healthcare professionals identified factors that would be helpful in implementing fall risk screening, including supportive staff and the attractiveness of the program. However, they were less positive than the patients and identified multiple factors as barriers, including their working style and their role perception. In particular, healthcare professionals who performed the screening identified many barriers.

Patients reported no barriers regarding patient characteristics, healthcare worker characteristics, context characteristics, or program characteristics for outpatient fall risk screening. However, there were some factors we did not look into in the current study that could be possible barriers. Factors that were not investigated in our study included transport problems and personal factors, and concerns including the risk of injuries, feeling too healthy, having impaired mobility, and trying something new, which were all barriers identified in other studies. Nevertheless, other studies have also mentioned barriers that were included in the current study, namely time investment and costs [16,32,33]. A possible explanation for the fact that the participants in the present study did not consider those practical factors to be barriers for participating in the outpatient fall risk screening is that the current study consisted only of screening and not of an intervention involving physical activity or additional appointments. In these interventions, costs and investment of time play a bigger role compared to a free screening during an appointment in which they had already invested time. Another barrier found in other studies is low motivation, due to a lack of perceived personal relevance. This was not observed in the current study, possible due to older adults’ health status during screening. Since all older adults were screened during their visit to a hospital, it can be assumed that they were experiencing health problems. Screening in an outpatient setting seems a great opportunity to make older adults more aware of their fall risk, as most patients in the present study indicated that they were more open to receiving advice on how to prevent falls after they were screened. The screening could be the first step in the prevention of falls, since older adults in our study were not only more open to advice, but also more inclined to take action to prevent falling.

Besides the time investment and costs, the attractiveness of the program and the concordance with cultural values (ethnicity) were also identified as facilitators in the current study. Patients reported expecting to benefit enough from the fall risk screening (attractiveness), which possibly made up for the small time and cost investments they had to make. In previous research, less focus has been placed on facilitators compared to barriers. In studies where facilitators have been reported, the interventions involved adjustments in the lives of older adults. Facilitators for these type of interventions include the perceived benefits, involvement of a healthcare professional, and social support [24]. As with barriers, we observed that this type of intervention involves different facilitators than the fall risk screening within an outpatient setting that we performed.

With respect to the second aim of this study, it was found that even though there were no significant differences within the statements, the acute-care patients considered more factors to be facilitators for participating in the screening program than chronic-care patients. This was mainly reflected in the healthcare worker domain by the statements “healthcare professionals do not have the right skills for fall risk screening” and “healthcare professionals do not have the right knowledge for fall risk screening”. On one hand, this difference between acute- and chronic-care patients might be noteworthy since chronic-care patients, due to their more regular visits, may feel a stronger bond with their physician. It could be argued that for that reason they have more trust in the actions of their physician. On the other hand, they may not be familiar with receiving care different from their usual care by their physician, which is not the case for acute-care patients. In addition, screening is more common at the ED than at other departments [33]. This was also reflected in the statement: “Fall risk screening belongs to the duties of nephrology/ED healthcare workers”, which was scored more positively by acute-care patients. In addition to the factor of familiarity, patients’ answers may have been affected by healthcare worker characteristics such as age, type of healthcare worker, experience, etc. However, since we did not collect these characteristics of healthcare providers for specific patients, we were not able to look into this possibility. Nevertheless, the fact that a lower trust in healthcare professionals with screening at the nephrology department did not lead to barriers among patients for implementing fall risk screening is a good starting point for further implementation in other departments.

Older adult patients perceived the outpatient clinic to be an appropriate place to be screened for fall risk. Healthcare professionals, however, were less positive. Although they considered several factors to be facilitators for implementing a fall risk screening program, they also perceived several factors that could inhibit the implementation of a fall risk screening program in an outpatient setting. Based on the different domains of the questionnaire, the program characteristics domain was reported most positively among healthcare providers. This implies that the setup of the program could be used in further implementation of outpatient fall risk screening interventions. In addition, healthcare providers who were involved in the screening stated that the program is handy for use, appropriate, and important. However, healthcare providers also stated that the program was useless and unwise, which indicated exceptionally low opinions of the program. Further research into the manner in which these factors influence healthcare providers’ motivations in the implementation of fall-prevention programs is necessary to fully understand this seeming discrepancy. A reason for the negative results might be the effort it involves for healthcare providers, which was also seen in overall barriers among healthcare providers. In particular, changing routines, resistance to working with protocols, and reading and remembering the fall-prevention program were considered impediments to implementing the screening program into practice. These barriers have not been directly observed in other studies, but they may be associated with a lack of motivation, which is a known barrier for implementing fall-prevention interventions [22]. Furthermore, other studies have reported financial situation, time investment, and patient health status as barriers [16,34]. A lack of knowledge is often found to be a barrier to implementation of fall risk interventions among healthcare professionals [22,34,35], but the findings of the current study did not confirm that. A possible explanation could be that the actual knowledge of healthcare professionals was not measured. The only two statements regarding knowledge did not involve general knowledge about fall prevention, but rather the wish to know more about the program and their own knowledge of preventive care. Another explanation could be that all healthcare professionals involved in the medical care of the older adults were included (e.g., nephrologist, emergency department specialist, nurse specialists), and the study was executed in a university medical center at which knowledge of healthcare professionals on evidence-based practice may be deemed to be sufficient [22].

In addition to the existing literature, this study helps to illustrate why cost-effective fall-prevention programs, such as fall risk screening, are not always successfully implemented. We knew that barriers were experienced with fall-prevention programs in community settings, and that these barriers were present among older adults as well as healthcare professionals. Unlike in prevention in community settings, this study showed that barriers within an outpatient setting are mainly reported by healthcare professionals and less among older adults. The barriers experienced were also different compared to the implementation of fall-prevention programs, and focused especially on the working style and attitude of professionals instead of their knowledge. However, it is not clear how healthcare professionals can overcome these specific barriers and how to increase intrinsic motivation among those professionals for successful implementation of fall risk screening. Studies using qualitative methods should look into this further, to find ways to overcome these barriers. Furthermore, it was not clear whether the characteristics of healthcare providers affected the responses of patients. Further research into the characteristics of healthcare providers could provide valuable insights related to the implementation of fall risk screening.

For future implementation of fall risk screening programs in outpatient settings, it is important to pay special attention to the barriers that were reported by the healthcare professionals. It could also be beneficial to enhance the facilitators that were described in the current study. Besides these facilitators, a perception of the usefulness of the intervention, satisfaction with medical care for fall and increased consultation time are associated with a better uptake of fall-prevention interventions [34]. However, despite the facilitators we found among healthcare professionals and patients, some facilitators had only a small majority, and this alone is not sufficient for successful implementation. Therefore, besides taking into account the barriers and facilitators from the current study, we recommend using existing implementation tools for the implementation of outpatient fall risk screening in daily practice. These implementation tools can help with informing and motivating healthcare professionals and increasing adherence to the screening guidelines [36]. Based on our experience with implementing the screening program, tools should focus on educating, informing, and motivating healthcare providers. Informing and educating could focus on changing outcome beliefs, which were low, especially among healthcare professionals who were involved in the screening. Only 21.1% agreed with the statement: “I believe that when I apply this fall-prevention program I can prevent falls”. Addressing issues about outcome beliefs and beliefs to make a difference are important for creating motivation and commitment [37]. In this case, especially a tool based on a capacity-building strategy, which targets motivation and capability to engage implementation processes [38], would be helpful. Capacity-building strategies can include internet-based instructions, training and workshops to increase knowledge, technical assistance, education using self-directed learning, communities of practice, and multi-strategy interventions [39]. Despite the fact that capacity-building strategies are usually applicable across multiple settings [38], further implementation research should focus on tools that can be used to overcome the specific barriers among healthcare providers for fall risk screening.

### Strengths and Limitations

This study had several strengths and limitations. It was performed in an outpatient setting, while previous research has looked more at barriers and facilitators in community settings. In addition, in the current study, we were able to find different barriers and facilitators for healthcare professionals that were involved in the same screening program at different time points. Another strength was that within this outpatient settings, we looked at both an acute care setting and a chronic care setting. Since these patients were in different conditions and had different reasons to visit the hospital, this could have led to different barriers and facilitators. At the same time, this study looked at patients’ as well as healthcare professionals’ barriers and facilitators.

When interpreting the results of the present study, it should be taken into account that only patients who participated in the screening program received a questionnaire to assess the barriers and facilitators they encountered for participating in the screening program. Older adults who declined the screening during their visit at the hospital might have had other replies, which could have resulted in more barriers being reported by patients. In addition, there was a risk of response (social desirability) bias, because older adults completed the questionnaire during their visit to the hospital. In addition to patients who declined screening, despite the 100 patients screened at the ED department, ultimately only 29 participated in the study. This could have influenced the results in the same way as mentioned above. Furthermore, we might have lost to follow-up the older adults who were less positive about fall risk screening and fall prevention in general. Therefore, we do have to be careful with interpreting the results of this acute-care group, but also in the comparison with chronic-care patients; acute-care patients were slightly more positive towards screening. In addition, we did not assess the characteristics of healthcare providers, which made it impossible to determine whether the characteristics of healthcare providers affected patients’ answers regarding the healthcare worker domain. Lastly, the questionnaire used was based on the “Barriers and Facilitators Assessment Instrument” developed by Peters et al., which is a validated questionnaire. However, for the current study, questions were removed and the Theory of Planned Behavior domain was added. This questionnaire has not been used and validated in previous research, and more research is necessary to validate the shortened questionnaire with the new domain for healthcare professionals, as well as the questionnaire used for patients.

## 5. Conclusions

It can be concluded that for older adults, the outpatient clinic and ED seems to be a good place to screen for fall risk. Most patients were positive about the non-invasive screening and willing to participate, which resulted in patients being more open to receiving fall-prevention advice. From that point of view, fall risk screening in these outpatient settings could be the first step to preventing falls among older adults by improving their fall risk awareness during their hospital visits. Healthcare professionals also reported factors that could enhance the implementation of outpatient fall risk screening. In particular, program characteristics were experienced as possible facilitators for implementation. However, implementation is complex and healthcare professionals reported barriers as well. The barriers that need to be addressed were especially related to the healthcare worker characteristics domain, involving a change of the mindset and routines of healthcare professionals. To achieve this, healthcare professionals need to be informed and motived to enhance intrinsic motivation. Existing implementation tools can also play an important role in this and should be used for successful implementation in daily practice of fall risk screening in outpatient settings.

## Figures and Tables

**Table 1:** Social demographics and risk of fall between acute-care and chronic-care patients.

	Total (n = 103)	Chronic Care (n = 74)	Acute Care (n = 29)	X^2^	*p*-Value
	N (%)	N (%)	N (%)		
Age in years, median (IQR)	74.0 (71.0–77.0)	73.0 (71.0–77.0)	75.0 (72.5–78.0)	854.5	0.107
**Gender**				0.384	0.535
Female	27 (26.7)	18 (25.0)	9 (31.0)		
Male	74 (73.3)	54 (75.0)	20 (69.0)		
**Ethnicity**				0.341	0.559
Dutch	87 (89.7)	62 (88.6)	25 (92.6)		
Other	10 (10.3)	8 (11.4)	2 (7.4)		
**Education level**				0.963	0.618
Low	48 (51.6)	37 (54.4)	11 (44.0)		
Intermediate	27 (29.0)	18 (26.5)	9 (36.0)		
High	18 (19.4)	13 (19.1)	5 (20.0)		
**Living situation**				4.637	0.098
Independent	84 (86.6)	62 (89.9)	22 (78.6)		
Independent with care	11 (11.3)	5 (7.2)	6 (21.4)		
Care institution	2 (2.1)	2 (2.9)	0 (0.0)		
**Living together**				0.687	0.407
Yes	75 (76.5)	52 (74.3)	23 (82.1)		
No	23 (23.5)	18 (25.7)	5 (17.9)		
**Housing type**				1.113	0.573
Ground floor	10 (10.3)	8 (11.6)	2 (7.1)		
House or flat with stairs	46 (47.4)	34 (49.3)	12 (42.9)		
House or flat with elevator	41 (42.3)	27 (39.1)	14 (50.0)		
**Chronic conditions**				3.198	0.074
At least one	81 (81.8)	55 (77.5)	26 (92.9)		
None	18 (18.2)	16 (22.5)	2 (7.1)		
**Elevated fall risk**				0.272	0.602
Yes	36 (35.0)	27 (36.5)	9 (31.0)		
No	67 (65.0)	47 (63.5)	20 (69.0)		
**Fall risk questions (Yes)**					
Fallen in the past year	36 (35.0)	24 (32.4)	12 (41.4)	0.734	0.392
Fallen multiple times	16 (15.5)	14 (18.9)	2 (6.9)	2.295	0.13
Problems moving	48 (46.6)	37 (50.0)	11 (37.9)	1.22	0.269
Afraid of falling	20 (19.8)	16 (22.2)	4 (13.8)	0.925	0.336

A *p*-value of <0.05 was considered statistically significant.

**Table 2:** Barriers and facilitators of chronic-care and acute-care patients considered in hospital fall risk screening.

		All Patients (n = 103)		Chronic Care (n = 74)		Acute Care (n = 29)		Chi-Square
**Negative statements**	Domain	agree	disagree	agree	disagree	agree	disagree	*p*-value
		%	%	%	%	%	%	
*Skills:* HCWs do not have the right skills for FRS.	HCW	16.5	44.7	18.9	39.2	10.3	**58.6**	0.190
*Knowledge:* HCWs do not have the right knowledge for FRS.	HCW	17.6	41.2	21.9	35.6	6.9	**55.2**	0.096
*Health status:* I do not appreciate it when people interfere with my health when I did not ask for it.	Patient	35.3	46.1	38.4	45.2	27.6	48.3	0.500
*Ethnicity:* Early detection of disease is not concordant with my culture/values.	Patient	11.0	**76.0**	8.3	**79.2**	17.9	**67.9**	0.359
*Financial status:* I am afraid FRS will cost me money.	Patient	19.0	**58.0**	22.5	**57.7**	10.3	**58.6**	0.252
*Compatibility:* I think FRS is executed at an inconvenient time.	Program	16.0	44.0	15.5	45.1	17.2	41.4	0.941
*Attractiveness:* I have negative expectations of FRS.	Program	11.9	**59.4**	12.5	**59.7**	10.3	**58.6**	0.922
*Specificity, flexibility:* I do not believe that FRS can detect fall risk at an early stage and prevent deterioration.	Program	33.3	32.4	34.2	31.5	31.0	34.5	0.941
*Didactive benefit:* Since I have been screened for fall risk, I am not going to prevent falling in a more active manner.	Program	28.2	30.1	25.7	29.7	34.5	31.0	0.578
**Positive statements**	Domain	agree	disagree	agree	disagree	agree	disagree	p-value
		%	%	%	%	%	%	
*Specificity, flexibility:* FRS belongs to the duties of nephrology/ED HCWs.	Program	48.0	16.7	42.5	19.2	**62.1**	10.3	0.191
*Attractiveness:* I expect to benefit from FRS.	Program	**59.8**	10.8	**54.8**	11.0	**72.4**	10.3	0.210
*Time investment:* I have enough time to be screened for fall risk.	Context	**75.2**	13.9	**75.3**	15.1	**75.0**	10.7	0.709
*Supportive staff:* There are enough staff at the nephrology/ED department to screen for fall risk.	Context	35.4	18.2	32.9	17.1	41.4	20.7	0.547
*Facilities:* The nephrology/ED department is an appropriate place for FRS.	Context	47.5	25.3	43.1	27.8	**59.3**	18.5	0.350
*Group norms, socialization:* People in my surroundings that are important to me would participate in FRS.	Context	40.6	15.8	36.1	13.9	**51.7**	20.7	0.121
*Motivation to change:* I feel a need to be screened for fall risk.	Patient	43.6	32.7	40.3	34.7	**51.7**	27.6	0.575
*Motivation to change*: Since I have been screened for fall risk, I am more aware of my risks of falling.	Patient	37.9	26.2	35.1	27.0	44.8	24.1	0.653

**Bold** = Facilitator for implementing the fall risk screening program. *Italic* = Indicates the subject of the statement. Domains are: HCW = healthcare worker characteristics; Patient = patient characteristics; Program = fall-prevention program characteristics; Context = context characteristics. Abbreviations used within statements: HCW = healthcare worker; FRS = fall risk screening; ED = emergency department. “Agree” means “agree” and “fully agree”. “Disagree” means “disagree” and “fully disagree”.

**Table 3:** Barriers and facilitators of healthcare providers to implementing the fall screening program in older adults.

		Screening (n = 19)		Follow up Advice (n = 17)	
**Negative statements**	Domain	agree	disagree	agree	disagree
		%	%	%	%
*Compatibility:* The fall-prevention program does not fit into my ways of working at my practice.	Program	36.8	21.1	11.8	5.9
*Time investment:* Working to the fall-prevention program is too time consuming.	Program	36.8	36.8	11.8	35.3
*Attitude, role perception:* I have a general resistance to working according protocols.	HCW	**78.9**	5.3	**52.9**	0.0
*Doubts about innovation:* I think parts of the fall-prevention program are incorrect.	HCW	47.4	5.3	47.1	0.0
*Lifestyle, working style:* I have problems changing my old routines.	HCW	**73.7**	15.8	**76.5**	0.0
*Education:* It is difficult to give preventive care because I am not trained in giving preventive care.	HCW	42.1	21.1	17.6	47.1
*Practice involvement:* I did not thoroughly read nor remember the fall-prevention program.	HCW	**78.9**	10.5	**52.9**	17.6
*Setup involvement:* It is difficult to give preventive care because I have not been involved in setting up the preventive care.	HCW	31.6	47.4	5.9	**64.7**
*Knowledge, motivation:* I wish to know more about this fall-prevention program before I decide to apply it.	HCW	36.8	21.1	29.4	**52.9**
*Ethnicity:* It is difficult to give this preventive care to patients with a different cultural background.	Patient	31.6	**57.9**	11.8	41.2
*Financial situation, economic status:* It is difficult to give this preventive care to patients with a low socioeconomic status.	Patient	**52.6**	26.3	17.6	35.3
*Number of patient contacts:* It is difficult to give this preventive care to patients who rarely visit the clinic.	Patient	**52.6**	26.3	5.9	**58.8**
*Health status:* It is difficult to give this preventive care to patients who seem healthy.	Patient	47.4	26.3	5.9	**58.8**
*Motivation to change:* Patients do not cooperate in applying this fall-prevention program.	Patient	**84.2**	5.3	29.4	17.6
*Group norms, socialization:* Colleagues from my discipline do not cooperate in applying the fall-prevention program.	Context	36.8	15.8	47.1	11.8
*Group norms, socialization:* Colleagues from other disciplines do not cooperate in applying the fall-prevention program.	Context	21.1	21.1	17.6	5.9
*Group norms, socialization:* Managers/directors do not cooperate in applying the fall-prevention program.	Context	**57.9**	5.3	35.3	0.0
*Reimbursement, insurance system:* Working according to this fall-prevention program requires financial compensation.	Context	15.8	21.1	11.8	5.9
*Opening hours of practice:* It is difficult to give preventive care because the timing of the preventative care is awkward.	Context	36.8	26.3	11.8	41.2
*Supportive staff:* It is difficult to give preventive care if there are not enough supportive staff.	Context	26.3	42.1	11.8	**76.5**
*Facilities:* It is difficult to give preventive care if instruments needed are not available.	Context	**63.2**	15.8	5.9	41.2
*Practice building:* It is difficult to give preventive care if physical space is lacking (e.g., rooms).	Context	**57.9**	21.1	23.5	47.1
*Attitude:* This fall-prevention program is useless.	TPB	**73.7**	5.3	29.4	11.8
*Attitude:* This fall-prevention program is unwise.	TPB	**68.4**	0.0	35.3	0.0
**Positive statements**	Domain	agree	disagree	agree	disagree
		%	%	%	%
*Specificity, flexibility:* This fall-prevention program leaves enough room for me to make my own conclusions.	Program	47.4	5.3	41.2	11.8
*Specificity, flexibility:* This fall-prevention program leaves enough room to weigh the wishes of the patient.	Program	47.4	5.3	47.1	5.9
*Didactive benefit:* This fall-prevention program is a good starting point for my self-study.	Program	15.8	21.1	47.1	29.4
*Attractiveness:* The layout of this fall-prevention program makes it handy for use.	Program	**63.2**	15.8	47.1	11.8
*Attitude:* This fall-prevention program is appropriate.	TPB	**73.7**	0.0	29.4	5.9
*Attitude:* This fall-prevention program important.	TPB	**63.2**	5.3	29.4	0.0
*Subjective norm:* Colleagues that I identify with would apply this fall-prevention program.	TPB	31.6	10.5	0.0	17.6
*Perceived behavioral control:* I believe that when I apply this fall-prevention program I can prevent falls.	TPB	21.1	15.8	**52.9**	5.9
*Intention:* I am willing to structurally apply this fall-prevention program to all my future patients aged 70 years and older.	TPB	36.8	21.1	5.9	23.5
*Behavior:* I have applied this fall-prevention program to patients in the past.	TPB	31.6	**63.2**	41.2	41.2

**Bold** is barrier or facilitator. *Italic* = Indicates the subject of the statement. Domains are: HCW = healthcare worker characteristics; Patient = patient characteristics; Program = fall-prevention program characteristics; Context = context characteristics; TPB = Theory of Planned Behavior.

## Appendix A

**Table A1:** Questionnaire for high-risk patients to assess perceptions about falls, fall risk, and fall prevention three months after screening in the hospital.

Statements	Totally Disagree	Disagree	Neutral	Agree	Totally Agree
1. I am more open to receiving advice on how to prevent falling.	□	□	□	□	□
2. I have a more positive attitude toward fall risk screening and fall prevention.	□	□	□	□	□
3. I am more inclined to take action to prevent falling.	□	□	□	□	□
4. I can count on more understanding and/or help from those around me to prevent falling.	□	□	□	□	□
5. I have more knowledge of actions I can take to prevent a fall.	□	□	□	□	□
6. I feel I am more self-sufficient.	□	□	□	□	□
7. I am more aware of the risk of falling.	□	□	□	□	□
8. I feel more capable of preventing a fall.	□	□	□	□	□
9. I did things to prevent falling.	□	□	□	□	□
